# Benzoic Acid, *Enterococcus faecium*, and Essential Oil Complexes Improve Ovarian and Intestinal Health via Modulating Gut Microbiota in Laying Hens Challenged with *Clostridium perfringens* and Coccidia

**DOI:** 10.3390/ani15030299

**Published:** 2025-01-21

**Authors:** Hongye Zhang, Fengjia Liang, Haojie Gong, Xiangbing Mao, Xuemei Ding, Shiping Bai, Qiufeng Zeng, Yue Xuan, Keying Zhang, Jianping Wang

**Affiliations:** Animal Nutrition Institute, Sichuan Agricultural University, Chengdu 611130, China; zhy1489529360@163.com (H.Z.); 13668363578@163.com (F.L.); ghjie2904@163.com (H.G.); acatmxb2003@163.com (X.M.); dingxuemei0306@163.com (X.D.); shipingbai@sicau.edu.cn (S.B.); zqf@sicau.edu.cn (Q.Z.); 71128@sicau.edu.cn (Y.X.); zkeying@sicau.edu.cn (K.Z.)

**Keywords:** *Clostridium perfringens* and coccidia, benzoic acid, *Enterococcus faecium* and essential oil complex, laying hens, intestinal health, gut microbiota

## Abstract

Intestinal disease is becoming increasingly prevalent in poultry production; however, the effect of BEC in laying hens challenged with *C. perfringens* and coccidia is limited. This study aimed to investigate the effects of dietary supplementation with BEC on intestinal and ovarian health in laying hens challenged with *C. perfringens* and coccidia. A total of 80 Lohmann gray hens (35 weeks) were randomly assigned to two dietary groups supplemented with BEC (0 or 1000 mg/kg). Each group contained 40 replicates, with one bird each (one hen per cage). During the sixth week of the trial, half of the laying hens in each group (*n* = 20) were administered 40 mL *C. perfringens* (2.5 × 10^10^ CFU/mL) and 0.15 mL coccidia (55,000 sporangia/mL), while the other half (*n* = 20) were administered 40 mL phosphate-buffered saline (PBS). The results indicated that those challenged with *C. perfringens* and coccidia had severely damaged jejunal and ovarian histopathological morphology, increased oxidative damage, decreased cecal acetic acid and butyric acid content (*p* < 0.05), and resulted in lower gut microbial richness and diversity. The diet of 1000 mg/kg BEC reduced the jejunal and ovarian pathological damage and oxidative damage, increased short-chain fatty acids (SCFAs) content, and enhanced gut microbial richness and diversity (*p* < 0.05) in laying hens challenged with *C. perfringens* and coccidia. Furthermore, the positive effects of BEC on intestinal health were associated with changes in gut microbial composition and structure. In summary, dietary supplementation with BEC has the potential to reduce the severity of intestinal and ovarian damage caused by challenges posed by *C. perfringens* and coccidia through the modulation of gut microbiota.

## Simple Summary

Prebiotics, organic acids, and plant essential oils are considered the effective additives in poultry feed. In this study, dietary benzoic acid, *Enterococcus faecium*, and essential oil complex (BEC) addition alleviated intestinal and ovarian pathological injury and oxidative damage in laying hens challenged with *Clostridium perfringens* (*C. perfringens*) and coccidia. BEC was attributed to improving microecological balance and intestinal redox balance by modulating gut microbial composition and structure.

## 1. Introduction

With the development of livestock husbandry worldwide, the production of poultry meat and eggs is constantly growing, and consumers are becoming increasingly demanding regarding the quality of these products [1]. However, gastrointestinal diseases are becoming increasingly prevalent in poultry and are considered to be the leading cause of serious economic loss to the poultry industry. One of the primary causes of intestinal disorders in chicken, including the well-known necrotic enteritis (NE) and coccidiosis caused by *C. perfringens* and coccidia, is the invasion and colonization by pathogenic microorganisms [2,3]. The proliferation of *C. perfringens* renders poultry vulnerable to coccidia, with NE and coccidiosis frequently coexisting [4]. Generally, infections of *C. perfringens* and coccidia in poultry are characterized by mental sluggishness, intestinal bleeding, diarrhea, a decline in feed intake and weight, and may even result in a significant number of deaths as the disease progresses [5,6]. More importantly, the pathogens from *C. perfringens* and coccidia not only negatively impact production performance and egg quality, but may also be deposited in poultry meat and eggs, endangering food safety and human health [7,8,9].

Antibiotics were extensively employed in poultry farming over 20 years ago with the intention of promoting growth, as well as curing and preventing illnesses. However, as time progresses, increasing evidence suggests the detrimental effects of antibiotic addition to chicken feed on both environmental and human health [10,11,12]. Consequently, the European Union explicitly prohibited the use of antibiotics in animal feed in 2006, and this regulation has been gradually implemented globally [13]. Following the ban on antibiotics, numerous studies have contributed to the development of effective alternatives in poultry feed that positively impact growth and production performance by enhancing nutrient utilization and improving intestinal health [14]. Due to their similar mechanisms to antibiotics, an increasing number of alternatives, including probiotics, prebiotics, organic acids, and essential oils, are being extensively used in poultry feed.

The aim of supplementing dietary alternatives in poultry feed is not only to resist invasion and infection by *C. perfringens* and coccidia but also to regulate gut microbiota, reduce the availability of pathogen receptors, protect the intestinal barrier, and consequently achieve a beneficial alteration in intestinal health, nutrient absorption, and production performance [15]. Recently, probiotics, prebiotics, organic acids, plant essential oils, and other additives have demonstrated the potential to provide a strong protective effect in chickens challenged with *C. perfringens* or coccidia [16,17,18]. Benzoic acid, *Enterococcus faecium*, and essential oils are natural compounds with positive roles in intestinal health, particularly in poultry. There is a study which indicated that pretreatment with *Enterococcus faecium* (2 × 10^8^ CFU/kg of diet ) could ameliorate NE in broilers [19]. A blend of encapsulated essential oils and organic acids (500 mg/kg) have also shown antimicrobial activity by lowering intestinal pH and enhancing intestinal immunity in broilers challenged with *C. perfringens* or coccidia [20,21]. Furthermore, combinations of benzoic acid, *Enterococcus faecium*, and essential oils (1000 mg/kg of diet) have been demonstrated in our earlier research to have positive effects on production performance and intestinal lesions in laying hens challenged with a combination of *C. perfringens* and coccidia [22,23]. It remains unknown whether the functional mechanism of BEC is linked to alterations in the gut microbiota community or whether it affects the ovarian health of laying hens. Therefore, the objective of this study was to investigate the effects of dietary BEC supplementation on the intestinal and ovarian health of laying hens challenged by *C. perfringens* and coccidia. Appropriate concentrations of *C. perfringens* and coccidia were administered to mimic the NE condition, to assess intestinal and ovarian histopathology assays, antioxidant abilities, cecal microbiota, SCFAs production, and related gene expression in the challenged laying hen, as well as to evaluate the role of BEC in alleviating intestinal and ovarian damage in challenged laying hens.

## 2. Materials and Methods

The commercial BEC product was purchased from DSM (DSM Nutritional Products Inc., Shanghai, China), and contained active constituents, including 70% benzoic acid (99.5% purity), 5% essential oils (thymol:carvacrol = 1:1), 5% *Enterococcus faecium* (2 × 10^8^ CFU/kg diet), and 20% carrier (50% silica and 50% dextrin). The strain of *C. perfringens* type A was obtained from the China Veterinary Drug Administration (CVCC2030). The strain was incubated in sterile thioglycolate liquid medium at 37 °C for 24 h after activation. The avian coccidiosis quadrivalent live vaccine was purchased from Foshan Standard Biotech Co., Ltd. (Guangzhou, China), and contained *Eimeria tenella*, *Eimeria maxima*, *Eimeria acevulina*, and *Eimeria giant*.

### 2.1. Experimental Birds and Study Design

A total of 80 35-week-old Lohmann gray hens were randomly assigned to two dietary groups supplemented with BEC at 0 mg/kg (CON) and 1000 mg/kg (BEC). Each group contained 40 replicates, with one bird each (one hen per cage). Prior to the onset of the feeding trial, a 7-day adaptation period was implemented. All hens were individually housed in a controlled environment room to maintain a temperature of approximately 22 °C and were subjected to a 16L:8D photoperiod. Hens were provided with free access to water and a complete feed, and the basal diets were formulated to meet the requirements of the National Research Council (NRC 1994) [24]. (Supplementary Table S1).

At the sixth week (42–48 d), half of the birds (*n* = 20) were randomly selected from the CON and BEC groups (designated as the CC and BECC groups, respectively) and received 20-fold anti-coccidia vaccine (0.15 mL, 55,000 coccidia sporangia/mL/hen) and 40 mL *C. perfringens* (2.5 × 10^10^ CFU /mL), while the remaining half (*n* = 20) received 40 mL PBS; the methods were identical to those used in our previous study [22]. Twenty-four hours after the last challenge, 10 hens from each group were selected for sampling. After sacrificing by cervical dislocation, about 1 cm of the mid-jejunum and ovary were dissected for histopathology analysis. The mid-jejunal mucosa and cecal contents were collected for antioxidant enzyme activities, gene expression, SCFAs content determination, and microbiome sequencing, respectively. The ovarian tissue (removing the histopathological sample) was obtained for antioxidant enzyme activities determination.

### 2.2. Histopathology Analysis and Score

The 1 cm^2^ sections from the middle jejunum and central section of the ovary were dissected, and used 10% paraformaldehyde for fixing 24 h, then removed from the stationary liquid, trimmed, washed, dehydrated, and embedded in paraffin. Sections with a thickness 4 μm were dewaxed in xylene, rehydrated, and stained with hematoxylin and eosin (H&E) as with the previous description [22]. The jejunal and ovarian histopathological images were obtained using an optical microscope (NIKON Eclipse Ci, Nikon Precision (Shanghai) Co., Ltd., Shanghai, China) and were analyzed with the NIKON Digital Sight DS-FI2 image system. The jejunum and ovary were scored for histopathology separately on a scale from 0 to 5. For the jejunum, 0 = normal tissue; 1 = slight mucosal epithelium integrity with minimal inflammatory infiltration; 2 = mucosal epithelium exfoliated with necrosis and mild inflammatory infiltration; 3 = mucosal epithelium exfoliated, lamina propria swollen, and heavy inflammatory infiltration; 4 = mucosal epithelium exfoliated, lamina propria swollen, and heavy inflammatory infiltration; 5 = mucosal epithelium was exfoliated and necrotic, with a large number of necrotic fragments in the intestinal lumen, lamina propria unclear, and extensive inflammatory infiltration [25]. For the ovary [26], 0 = normal tissue; 1 = complete follicular structure with a few heterophilic granulocytes; 2 = mild perifollicular hemorrhage, cytoplasmic eosinophilic enhancement with foamy macrophage infiltration; 3 = follicular structure deformed, nuclear pycnosis and hyperstaining, fragmentation, or dissolution with excessive infiltration of foam cells; 4 = follicular structure severely deformed, with small necrotic areas, numerous heterophilic granulocytes, and nuclear fragmentation; 5 = follicular structure blurred, with significant nuclear fragmentation, massive necrosis, and fusion into a sheet of unstructured eosinophilic substance.

### 2.3. Measurement of Antioxidant Capacity in Jejunal Mucosa and Ovary

Jejunal mucosa and ovary tissues were used to determine antioxidant capacity. The activities of superoxide dismutase (SOD), total antioxidant capacity (T-AOC), catalase (CAT), glutathione peroxidase (GSH-Px), glutathione s-transferase (GSH-ST), glutathione (GSH), and malondialdehyde (MDA) concentrations were measured using commercial kits (Nanjing Jiancheng Biotechnology Co., Ltd., Nanjing, China) with enzyme-linked immunosorbent assay.

### 2.4. Real-Time PCR for Jejunal Antioxidant-Related mRNA Expression

Total RNA was extracted using TRIzol reagent (TaKaRa, Beijing, China) from frozen jejunal mucosa samples in accordance with the manufacturer’s instructions. The concentration of total RNA was determined using a DU 640 UV spectrophotometer (Beckman Coulter Inc., Fullerton, CA, USA), and reverse transcription reactions were immediately performed using the PrimeScript RT Reagent Kit (Takara). The cDNA was used for gene expression analysis using SYBR green qPCR master mix (Takara, Dalian, China). The amplification conditions were as follows: 95 °C for a duration of 15 s, followed by 40 cycles of 95 °C for 30 s, and 60 °C for 34 s with a final melting curve analysis. Real-time PCR for measuring gene expression was performed on the ABI QuantStudio^TM^ 6 Flex system (Applied Biosystems, Waltham, MA, USA). The primer sequences for all target genes (Nrf2, Keap1, NQO1, HO-1, NF-κB, p53) are shown in Supplementary Table S2. Each sample was assayed in triplicate, and relative gene expression was quantified by normalizing to avian β-actin and calculated using the 2^−ΔΔCt^ method.

### 2.5. Cecal SCFAs Analysis

Cecal digesta samples were diluted with ultrapure water to achieve uniformity and then centrifuged at 3000× *g* for 15 min. The supernatant was collected immediately and mixed with ice-cold 25% (*w*/*v*) metaphosphoric acid solution, then incubated at 4 °C for 30 min and centrifuged at 11,000× *g* for 15 min. The SCFAs, including acetic acid (AA), propanoic acid (PA), butanoic acid (BA), isobutyric acid (IBA), valeric acid (VA), and isovaleric acid (IVA), were separated and analyzed using the Agilent 6890 gas chromatograph (Agilent Technologies, Santa Clara, CA, USA).

### 2.6. Gut Microbiota Sequencing Analysis

Total microbial genomic DNA was extracted from each sample using the QIAamp Fast DNA Stool Mini Kit (QIAGEN China (Shanghai) Co., Ltd, Shanghai, China) according to the manufacturer’s instructions. The concentration of DNA was determined using an enzyme standard instrument (Multiskan^TM^ GO). PCR amplification of the V3-V4 variable regions of 16S rDNA was performed to generate an amplicon sequence library by using specific primers 515F (5′-GTGCCAGCMGCCGCGGTAA-3′) and 806R (5′-GGACTACHVGGGTWTCTAAT-3′). The paired-end sequencing was performed using the Illumina HiSeq platform (Novogene Biotech Co., Ltd., Beijing, China). The raw sequencing reads were demultiplexed, and a quality filter was applied using the fastp. The reads were then merged using FLASH to obtain high-quality effective tags. This process was then subject to a quality control process of QIME (version 1.17). Subsequently, the remaining high-quality sequences were subjected to clustering into operational taxonomic units (OTUs) with a similarity level of 97%, utilizing the UPARSE software (v7.0.1090).

### 2.7. Statistical Analysis

The data were analyzed using the Shapiro–Wilk and Levene’s tests to assess normal distribution and homogeneity of variances. Normally distributed datasets were statistically analyzed using a two-way analysis of variance (ANOVA) with the General Linear Model (GLM) program, followed by Tukey’s test for multiple comparisons using SAS 9.2 software (SAS Institute, Cary, NC, USA). The main effects of the included challenge and dietary BEC level, as well as their interaction, were determined. The microbiota data were analyzed using the Wilcox rank sum test, and Beta diversity based on the weighted UniFrac distance matrices were calculated with QIIME (Version 1.7.0). Cluster analysis was performed using principal components analysis (PCA). Differentially represented bacterial taxa between samples were analyzed using the linear discriminant analysis effect size (LEfSe). Spearman’s correlation analysis was employed to explore the correlation between gut microbiota and several important indices. Differences with *p* < 0.05 were considered significant.

## 3. Results

### 3.1. Histopathological Scores and Examination of Intestine and Ovary

**Figure 1:**
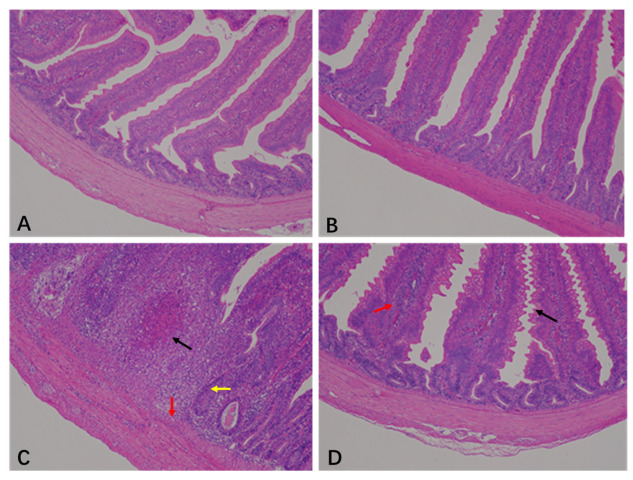
Histopathology analysis in the jejunum of laying hens. (**A**) There were no obvious histopathological lesions in the jejunum of the CON group. (**B**) The structure was clear and complete, with no necrotic lesions in the jejunum of BEC group. (**C**) The jejunum section appeared with necrotic lesions (black arrow), mucosal cell necrosis and inflammatory cell infiltration (red arrow), and intestinal glands atrophy (yellow arrow) from the CC group. (**D**) In the jejunum of the BECC group, serrated processes were found in the villous epithelium (black arrow), and lamina propria swelling accompanied by inflammatory cell infiltration (red arrow). Magnification: 100×.

The histopathological scores of the jejunum are shown in Table 1 and Figure 1. The jejunal histopathology scores from the CON and BEC groups were 0.5 and 0.38, respectively. The jejunal histopathology showed a clear and intact epithelial structure with villi arranged neatly (Figure 1A,B). The morphology and mucosal barrier were severely damaged by *C. perfringens* and coccidia, resulting in the exfoliation and necrosis of villus epithelial cells, variable infiltration of inflammatory cells in the epithelium and lamina propria, and necrotic lesions in the mucosa. The score was 4.5 (Figure 1C). In contrast, dietary supplementation with BEC alleviated jejunal injury, as indicated by slight changes in villus morphology and inflammatory cells infiltration (Figure 1D), resulting in a score of 3.25. Dietary supplementation with BEC significantly improved jejunal pathological conditions of laying hens challenged with *C. perfringens* and coccidia (*p* < 0.05).

**Figure 2:**
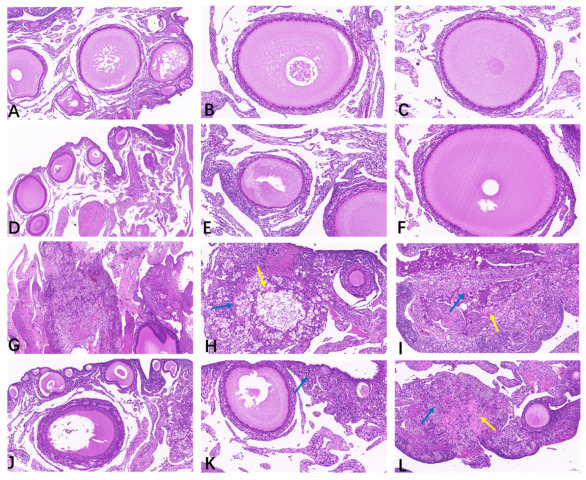
Histopathology analysis of ovarian tissues. (**A**–**C**) The follicles were arranged neatly, and the structure was clear in the ovarian tissue of the CON group. (**D**–**F**) The morphology of follicles was smooth, and granulosa cells were in normal arrangement; there were no obvious histopathological lesions observed in the ovary from the BEC group. (**G**–**I**) Massive necrosis of ovary tissue from the CC group, loss of normal oocyte and granulosa cell structure in the follicle, a growing number of parenchymal cell apoptosis, nuclear condensation, fragmentation or dissolution, and there appeared a large amount of eosinophil (yellow arrow) accompanied by foam cells infiltration (blue arrow). (**J**–**L**) The follicles exhibited abnormal morphology, a small area of tissue necrosis, cytoplasmic eosinophilic enhancement (yellow arrow), and a small amount of foam cells infiltration (blue arrow) in part of ovarian interstitial from the BECC group.

As illustrated in Table 1 and Figure 2, the ovaries from the CON (Figure 2A–C) and BEC (Figure 2D–F) groups were histologically uniform with no obvious abnormalities, exhibiting complete and clear morphology and structure of oocytes and follicles, with granulosa cells arranged neatly. The ovarian scores from the CON and BEC group were 0.25 and 0.5, respectively. The ovaries from the CC (Figure 2G–I) and BECC (Figure 2J–L) groups were significantly affected by *C. perfringens* and coccidia, resulting in massive necrosis of the ovarian tissue, numerous nuclei becoming pycnotic and undergoing karyorrhexis and karyolysis, and loss of the normal oocyte and follicle structure, accompanied by foamy macrophage infiltration in the stroma and cytoplasmic eosinophilic enhancement. Compared to the no-challenge groups, the ovaries from the challenge groups had a higher histopathology score (*p* < 0.05), and dietary supplementation with BEC significantly reduced the severity of ovarian damage caused by the challenges.

### 3.2. Intestinal and Ovarian Antioxidant Capacities

The activities of antioxidant enzymes and the content of oxidation product in the jejunum are presented in Table 2. The *C. perfringens* and coccidia challenge significantly enhanced the activities of SOD, CAT, GSH, and GSH-ST, as well as MDA concentration (*p* < 0.05). The antioxidant capacity of jejunum in the BEC group did not exhibit significant differences compared to the CON group. Dietary supplementation with BEC in laying hens challenged with *C. perfringens* and coccidia significantly decreased MDA concentration (*p* < 0.05).

The antioxidant-related mRNA expressions in the jejunum are illustrated in Table 3. The expression levels of nuclear factor erythroid 2-related factor 2 (*Nrf2*), NAD(P)H: quinone oxidoreductase 1 (*NQO1*), and tumor protein p53 (*p53*) in the jejunum were significantly higher in the BEC group (*p* < 0.05). However, the CC group significantly downregulated the expression level of Kelch-like ECH-associated protein (*Keap1*) (*p* < 0.05) and tended to reduce the expression level of *p53* (*p* = 0.06). Additionally, no significant interactions were observed between the CC group and the BEC group regarding the expressions of jejunal antioxidant-related genes.

The antioxidant capacity of the ovary was further analyzed (Table 4). The results of the antioxidant enzyme and oxidation product analyses indicated that *C. perfringens* and coccidia challenge had an effect on CAT activity (*p* = 0.02) and significantly increased MDA level (*p* < 0.05). The reduction of antioxidant capacity was not normalized by dietary supplementation with BEC, and no significant interaction was observed (*p* ≥ 0.05).

### 3.3. SCFAs Concentrations of Cecal Digesta

**Figure 3:**
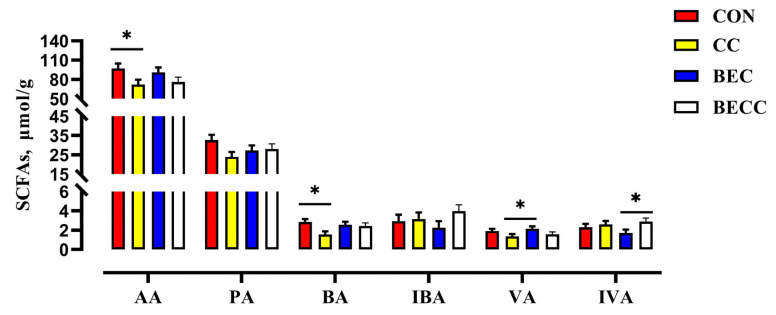
The effect of *C. perfringens* and coccidia and dietary BEC supplementation on the cecal SCFAs. Each mean represents one layer/replicate, eight replicates/treatment. CON, control group; CC, *C. perfringens* and coccidia challenge; BEC, basal diet + 1000 mg/kg BEC; BECC, *C. perfringens* and coccidia challenge + 1000 mg/kg BEC; acetic acid (AA), propionic acid (PA), butyric acid (BA), isobutyric acid (IBA), valeric acid (VA), isovaleric acid (IVA). Data are represented by vertical bars or plot individual values, * *p* < 0.05. CON = control group, CC = *C. perfringens* and coccidia challenge, BEC = basal diet + 1000 mg/kg BEC, BECC = *C. perfringens* and coccidia challenge + 1000 mg/kg BEC.

The composition and concentrations of SFCAs in the cecal digesta of laying hens are shown in Figure 3. The *C. perfringens* and coccidia challenge significantly decreased the concentrations of acetic acid and butyric acid (*p* < 0.05). No significant changes were observed in the main SFCAs with dietary BEC addition. In the laying hens challenged with *C. perfringens* and coccidia, the dietary addition with BEC reversed the reductions in the concentrations of acetic acid, propionic acid, butyric acid, and valeric acid.

### 3.4. Composition and Structure of Cecal Microbial Community

**Figure 4:**
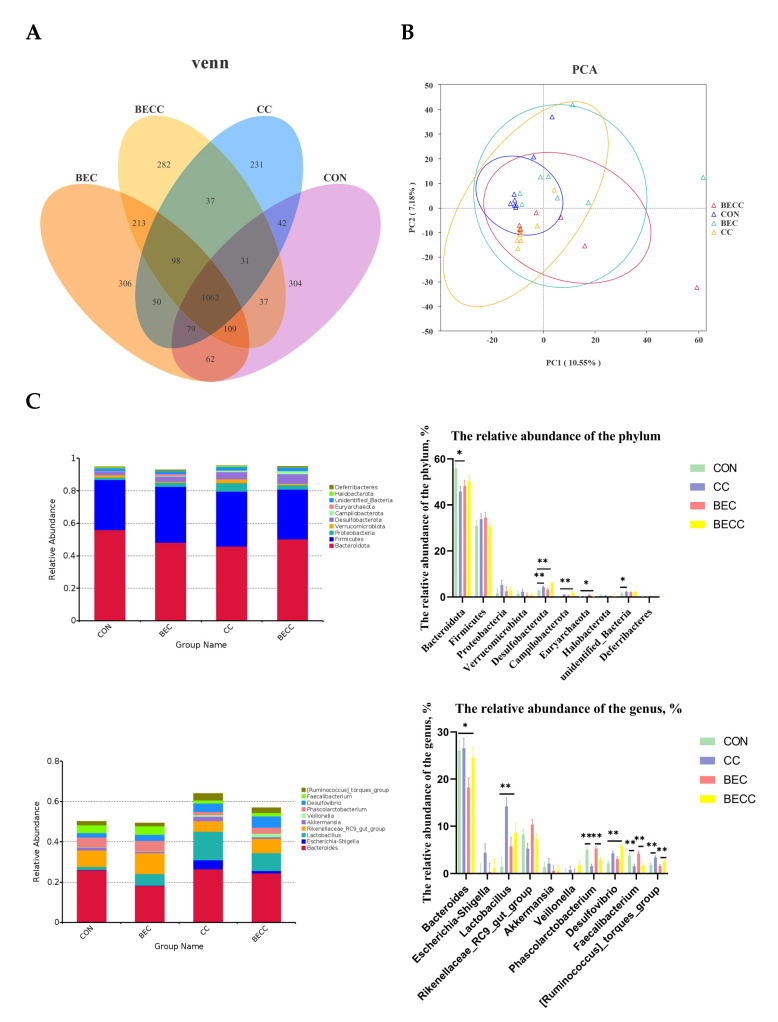
Cecal microbial composition and structure of laying hens. (**A**) Venn diagrams for bacterial OTUs. (**B**) The principal components analysis (PCA) plots to compare beta diversity of cecal microbiota. (**C**) The relative abundance of the top 10 phylum and genus from all groups. Each mean represents one layer/replicate, eight replicates/treatment. CON, control group; CC, *C. perfringens* and coccidia challenge; BEC, basal diet + 1000 mg/kg BEC; BECC, *C. perfringens* and coccidia challenge + 1000 mg/kg BEC. * means *p* < 0.05, ** means *p* < 0.01.

The cecal contents collected from all treatment groups were analyzed to determine microbial composition and structure. There were 304, 306, 231, and 282 unique operational taxonomic units (OUTs) in the CON, BEC, CC, and BECC groups, respectively (Figure 4A). As shown in Table 5, the alpha diversity of the microbial community, Shannon and Simpson indices, were significantly reduced (*p* < 0.01) by the *C. perfringens* and coccidia challenge, but there were no significant differences for the Chao 1 and ACE indices. Dietary supplementation with BEC had a positive effect on alpha diversity. However, the *C. perfringens* and coccidia challenge and the addition of BEC showed no significant interaction. The PCA plots of microbial community beta diversity indicated that the microbial community structure changed with different treatments (Figure 4B). The results revealed that the *C. perfringens* and coccidia challenge, along with the addition of BEC, altered the beta diversity of the microbial community. However, the occupied positions of the microbial community were partially overlapped in all treatment groups.

Figure 4C illustrated the cecal microbial composition at the phylum and genus levels and displayed the relative abundances of the top 10 phyla or genera. *Bacteroidota* and *Firmicutes* were the dominant phyla, accounting for approximately 80% of all groups. In laying hens challenged with *C. perfringens* and coccidia, a lower abundance of *Bacteroidota* and higher abundances of *Desulfobacterota* and *Campilobacterota* were observed (*p* < 0.05), whereas the abundance of these microbiotas was reversed by dietary supplementation with BEC. At the genus level, *Bacteroides* was the most dominant bacteria in all groups. *C. perfringens* and coccidia markedly altered the abundance of *Lactobacillus*, *Phascolarctobacterium*, *Desulfovibrio*, and *Faecalibacterium* (*p* < 0.01). In addition, the dietary supplementation with BEC resulted in a lower abundance of *Bacteroides* (*p* < 0.05).

**Figure 5:**
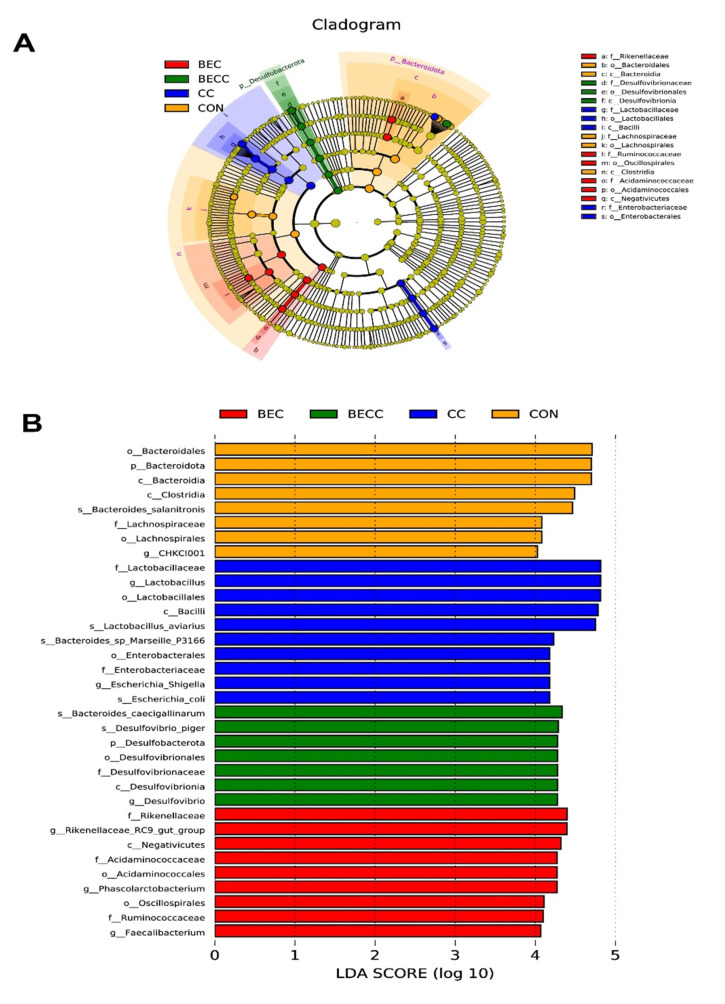
Linear discriminant analysis effect size (LEfSe) analysis shows the taxonomic profiling from phylum to genus levels. (**A**) Biomarker taxa are heighted by colored circles and shaded areas. Each circle’s diameter is relative to the abundance of taxa in the community. (**B**) Only taxa meeting an LDA score > 4 are shown. (Red) BEC-enriched taxa; (green) BECC-enriched taxa; (blue) CC-enriched taxa; (orange) CON-enriched taxa. Each mean represents one layer/replicate, eight replicates/treatment. CON, control group; CC, *C. perfringens* and coccidia challenge; BEC, basal diet + 1000 mg/kg BEC; BECC, *C. perfringens* and coccidia challenge + 1000 mg/kg BEC.

A further linear discriminant analysis effect size (LEfSe) was conducted to identify specific microbiome profiles and filter different biomarkers associated with all groups (*p* < 0.05; LDA > 2.0). The results displayed 8, 10, 7, and 9 different bacterial taxa in the CON, CC, BEC, and BECC groups, respectively (Figure 5). Obviously, the *C. perfringens* and coccidia challenge and dietary supplementation with BEC strongly affected microbial community composition at all levels, from the phylum to genus. In non-challenged laying hens, a large abundance of *Bacteroidota* (*Bacteroidia*, *Bacteroidles*, and *Bacterodies_salanitronis*), *Lachnospirales* (*Lachnospiracea*), and *Lactobacillales* was observed in the CON group. The BEC group exhibited a higher abundance of *Desulfobacterota* and subordinate bacteria. After the challenge, the CC group significantly increased the relative abundance of order *Lactobacillales*, family *Enterobacteriaceae*, and their subordinate bacteria, the BECC group enriched numerous bacterial members, including class *Negativicutes*, order *Acidaminococcales* (*Acidaminococcaceae*) and *Oscillospirales*, family *Rikenellaceae* and *Ruminococcaceae*, and genus *Phascolarctobacterium*, *Faecalibacterium*, and *Rikenellaceae_RC9_gut_group*.

### 3.5. Correlation Between the Intestinal Health and Cecal Microbiota

**Figure 6:**
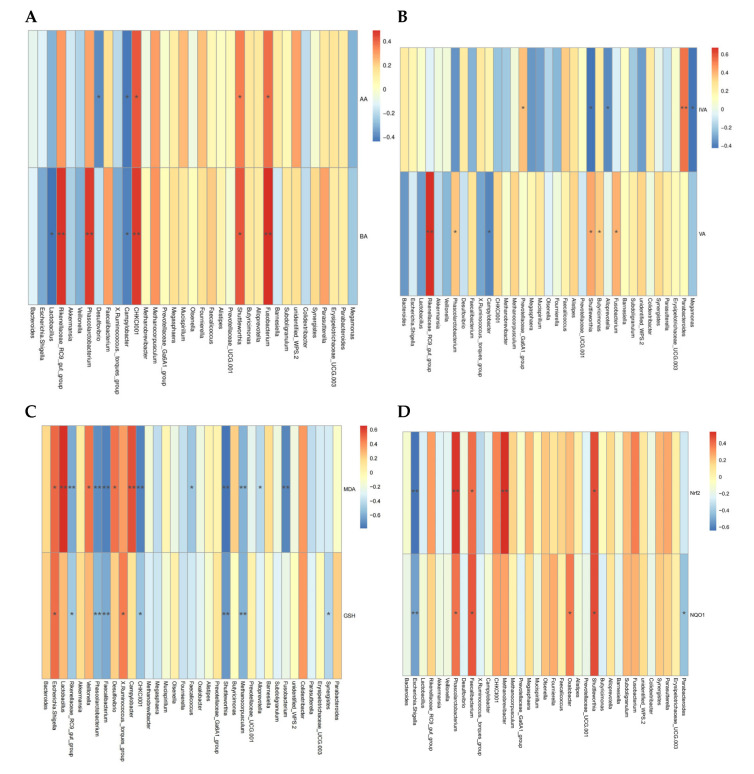
Heatmap of Spearman’s correlations between the gut microbiota at genus level (Top 35) and intestinal metabolite and intestinal antioxidant capacity. (**A**,**B**) Correlation between the microbiota and cecal SCFAs. (**C**,**D**) Correlation between the antioxidant enzyme activity, peroxidation product, and related-gens and the microbiota. Red indicates a positive correlation, and blue indicates a negative correlation; while the color is darker, the correlation is higher. * *p* < 0.05, ** *p* < 0.01. Each mean represents one layer/replicate, eight replicates/treatment. CON, control group; CC, *C. perfringens* and coccidia challenge; BEC, basal diet + 1000 mg/kg BEC; BECC, *C. perfringens* and coccidia challenge + 1000 mg/kg BEC.

To evaluate the deeper relationship between altered bacterial biomarkers and jejunum health-related indices, we correlated these variable data with microbiota at the genus level and conducted Spearman’s correlation analysis (Figure 6). Cecal acetic acid was positively correlated with *Fusobacterium*, *Shuttleworthia*, and CHKCI001 (*p* < 0.05, r > 0.5), but negatively correlated with *Campylobacter* and *Desulfovibrio* (*p* < 0.05, r < −0.5). Cecal butyric acid was positively correlated with *Fusobacterium*, *Shuttleworthia*, *Phascolarctobacterium*, and *Rikenellaceae_RC9_gut_group* (*p* < 0.05, r > 0.5), but negatively correlated with *Lactobacillus* and *Campylobacter* (*p* < 0.05, r < −0.5). Cecal valeric acid was positively correlated with *Fusobacterium*, *Butyricimonas*, *Shuttleworthia*, and *Rikenellaceae_RC9_gut_group* (*p* < 0.05, r > 0.5), but negatively correlated with *Campylobacter* (*p* < 0.05, r < −0.5). Cecal isovaleric acid was positively correlated with *Parabacteroides* (*p* < 0.05, r > 0.5), and negatively correlated with *Shuttleworthia*, *Megamonas*, and *Alloprevotella* (*p* < 0.05, r < −0.5). The MDA concentration of jejunum had a positive correlation with *Lactobacillus, Campylobacter*, *Desulfovibrio*, *Veillonella*, and *Escherichia-shigella* (*p* < 0.05, r > 0.5), but a negative correlation with *Fusobacterium*, *Methanocorpusculum*, *Shuttleworthia*, *CHKCI001*, *Faecalibacterium*, *Phascolarctobacterium*, and *Rikenellaceae_RC9_gut_group* (*p* < 0.05, r < −0.5). The GSH activity of jejunum had a positive correlation with *Escherichia-shigella*, and *X. Ruminococcus* (*p* < 0.05, r > 0.5), but a negative correlation with *Phascolarctobacterium*, *Faecalibacterium*, *Shuttleworthia*, and *Methanocorpusculum* (*p* < 0.05, r < −0.5). Furthermore, *Shuttleworthia*, *Phascolarctobacterium*, and *Faecalibacterium* were positively correlated with the expression levels of *Nrf2* and *NQO1* (*p* < 0.05, r > 0.5), and *Methanocorpusculum* was positively correlated with Nrf2 (*p* < 0.05, r > 0.5). However, *Escherichia-shigella* was negatively correlated with *Nrf2* and *NQO1* (*p* < 0.05, r < −0.5).

We observed that certain specific microbial biomarkers (e.g., *Escherichia-shigella*, *Shuttleworthia*, *Phascolarctobacterium*, *Faecalibacterium*, and *Campylobacter*) have a strong association with intestinal metabolites and antioxidant capacity.

## 4. Discussion

NE and coccidiosis represent persistent challenges that cause enormous financial losses alongside substantial expansion in the global chicken industry, particularly following the implementation of restrictions on antibiotics in diets [3,27]. Since *C. perfringens* (include type A, C, and G) can produce various toxins and is associated with coccidial infections, it is regarded as the major pathogenic strain of NE [28]. An increasing number of studies, including our previous research, have indicated that intestinal damage caused by *C. perfringens* and coccidia inhibits nutrient absorption and decreases productive performance in domestic birds [20,23,29]. These findings highlight the urgent need to fully understand the biological threats posed by *C. perfringens* and coccidia and to develop effective products for poultry feed. Traditional strategies for preventing and treating NE have relied on antibiotics (e.g., lincomycin, tylosin, and bacitracin). Nevertheless, a growing number of countries have prohibited the use of antibiotics in animal feeds, thereby encouraging environmentally friendly agricultural practices [14]. With advancements in feed science, more research has demonstrated that organic acids, probiotics, and essential oils serve as effective alternatives to antibiotics, improving intestinal health and productivity of poultry [15,19,30]. These antibiotic alternatives also have significant potential to control and mitigate NE [20,21,31,32]. Notably, our previous study demonstrated that the addition of benzoic acid, essential oils, and Enterococcus faecium significantly improved intestinal barrier integrity, inflammatory response, and egg performance in laying hens challenged with *C. perfringens* and coccidia [22]. It is hypothesized that these results are closely linked to the enhanced intestinal and ovarian health observed in the challenged laying hens as a consequence of BEC.

Typically found in the intestine of domestic birds, *C. perfringens* may proliferate rapidly by utilizing intestinal resources [33]. More frequently, it damages the normal function of the intestines in conjunction with coccidia [18], indicating that its pathogenic potential is closely linked to coccidiosis. Previous studies have confirmed that *C. perfringens* and coccidia can cause NE in birds, manifesting concretely as changes in intestinal morphology, including a reduced villus height to crypt depth ratio, decreased digestive enzyme activity, the release of inflammatory cytokine, and intestinal barrier injury [34,35]. The current study found similar results, showing that laying hens challenged with *C. perfringens* and coccidia exhibited significant pathological alternations and jejunal morphological damage. Furthermore, dietary supplementation with BEC exerted significant amelioration of intestinal morphology and lesions in laying hens challenged with *C. perfringens* and coccidia. Pham et al. [21] demonstrated that the addition of a combination of essential oils and organic acids to broiler diets repaired intestinal damage caused by the NE challenge. Levkut et al. [36] found that intestinal morphology improved when broilers challenged with *Salmonella Enteritidis* were fed *Enterococcus faecium* supplementation. This evidence demonstrated that BEC positively impacts the preservation of intestinal integrity and the reduction of histopathological lesion scores, likely by suppressing the proliferation of intestinal pathogens [37]. In our study, ovarian morphology and pathological conditions were significantly worse, supporting previous observations that pathogenic bacteria can survive and colonize in the ovaries of challenged birds [16]. We found that the addition of BEC also improved ovarian lesions, the morphology of the follicles, and the arrangement of granulosa cells were restored to some degree. The findings were consistent with our previous study [22], which indicated that dietary supplementation with BEC could increase egg production and improve egg quality in challenged laying hens by suppressing pathogenic bacteria in the ovary and protecting normal follicular development.

The redox balance plays an important role in animal health. Reactive oxygen species (ROS) are necessary for cell activities, but excessive accumulation of ROS can cause a dynamic imbalance between the oxidant and antioxidant systems when animals are exposed to environmental toxicants and other stressors [38]. The activities of antioxidant enzymes (including SOD, CAT, GSH, GSH-PX, GSH-XT, T-AOC) as well as the levels of oxidative products are considered key indices for evaluating the level of oxidative stress [39]. Prior research using poultry NE models indicated that birds challenged with lipopolysaccharides (LPS) and *Escherichia coli* produced more ROS, had higher intestinal MDA levels, and exhibited poorer activities of SOD, GSH-PX, and T-AOC [40]. In our study, *C. perfringens* and coccidia elevated SOD, CAT, GSH-ST, and T-AOC activities, as well as robustly induced MDA generation in jejunum, suggesting that NE challenge rapidly activates the antioxidant defense system to eliminate excessive ROS and minimize intestinal oxidative damage [41]. Meanwhile, the expression levels of oxidative stress-related mRNAs in the jejunal mucosa changed due to redox system imbalance. The current study showed that challenged laying hens had lower expression levels of *Nrf2*, *Keap1*, *NQO1*, and *OH-1*, which are the important pathway signals for counteracting oxidative stress [42]. These results indicate that *C. perfringens* and coccidia induced intestinal oxidative stress in laying hens, although the challenge is resisted by activating antioxidant enzymes. However, it ultimately fails to restore redox balance. Furthermore, we found that the addition of BEC decreased MDA levels and upregulated the expression of *NQO1* in the jejunum of laying hens challenged with *C. perfringens* and coccidia. The literature indicates that the benzoic acid, essential oils, and Enterococcus faecium exhibit antioxidant activity by scavenging ROS and free radicals, thereby maintaining intestinal redox homeostasis [43]. In addition, *C. perfringens* and coccidia significantly increased MDA levels in the ovaries, but dietary BEC supplementation had no significant effect on relieving oxidative damage, indicating that BEC is predominantly utilized in the intestine, resulting in differential alleviation of oxidative stress in the intestine compared to the ovary.

Gut microbiota and their metabolites play a crucial role in responding to intestinal inflammation and maintaining intestinal homeostasis, which is strongly correlative with nutrient digestion, absorption, utilization, and overall poultry production [44]. To investigate the relationship between microbiota and intestinal health, we analyzed the cecal microbial composition and structure. The current study found that *C. perfringens* and coccidia significantly reduced cecal microbial richness and diversity. Conversely, dietary supplementation with BEC significantly increased cecal microbial richness and diversity in laying hens challenged with *C. perfringens* and coccidia. Beta diversity analysis indicated that both BEC and the challenges significantly altered microbial community structure, consistent with previous study [21]. Higher intestinal microbial diversity is indicative of a more stable microbial community [45], suggesting that dietary BEC not only effectively improves the microbial diversity of normal laying hens but also impacts the composition of microbial communities in challenged laying hens. Further analysis of the relative abundance of dominant microbiota at both the phylum and genus levels was conducted to gain a deeper understanding of microbial community structure. The results indicated that *Bacteroidota*, *Firmicutes*, and *Proteobacteria* were the primary dominant bacteria at the phylum level, with a consistent increase in the abundance of *Bacteroidota* and a decrease in *Firmicutes* and *Proteobacteria* following BEC dietary intervention in challenged hens compared to those on a basal diet. *Bacteroidota* and *Firmicutes* are essential for maintaining intestinal homeostasis, being involved in nutrient metabolism and immune modulation [46]. In contrast, *Proteobacteria* is considered a significant inflammation-driven bacteria in the intestine [47]. Our results suggest that dietary BEC could alleviate intestinal inflammation in laying hens by increasing the relative abundance of *Bacteroidota* and inhibiting the growth of *Proteobacteria*. Moreover, in challenged laying hens, BEC supplementation decreased the abundance of *Lactobacillus* and *Escherichia-shigella*, while increasing the abundance of *Phascolarctobacterium*. *Lactobacillus* is recognized as a probiotic within *Firmicute*, capable of colonizing the intestinal surface of the host, producing antimicrobial peptides and bacteriocins to modulate intestinal microbiota and participate in immunomodulatory responses [48]. Conversely, *Escherichia-shigella* has been widely recognized as a negative indicator in intestinal microbiota, particularly in birds with severe intestinal inflammation [17]. Notably, the expansion of *Escherichia-shigella* can reduce the relative abundance of beneficial bacteria [49], and our results are consistent with this observation. Additionally, a reduction in the relative abundance of *Phascolarctobacterium* was observed in laying hens with NE; this bacterium is crucial for fermentation processes that produce SCFAs, such as acetic acid, butyric acid, and propionic aid [50]. Importantly, dietary BEC supplementation increased the relative abundance of *Phascolarctobacterium* in challenged laying hens, correlating with elevated SCFA concentrations in cecal contents. SCFAs, as microbiota-derived metabolites, provide energy for enterocyte in the cecum or colon, help inhibit intestinal inflammatory response, and participate in the modulation of intestinal homeostasis [51]. Consequently, the variations in the relative abundances of these dominant microbial taxa may be key factors determining the fate of intestinal inflammation in laying hens challenged with *C. perfringens* and coccidia, as well as the subsequent alleviating effects of diet BEC.

To clearly understand the role of intestinal microbial communities, LEfSe analysis and Spearman’s correlation analysis further identified biomarker microbiotas and the relationship with intestinal redox homeostasis. In challenged laying hens, *Lactobacillales* (*Lactobacillaceae*, *Lactobacillus*, and *Lactobacillus_aviarius*), *Enterobacterales* (*Enterobacteriaceae*), *Escherichia-shigella*, and *Escherichia_coli* were enriched as biomarkers. All of these have been reported to be associated with intestinal inflammation except for *Lactobacillales* [49,52]. Although *Lactobacillales* is considered an intestinal probiotic, its rapid increase can compete for nutrients and exacerbate the intestinal inflammatory response during pathogenic bacteria infection [53]. Dietary supplementation with BEC showed that *Phascolarctobacterium*, *Ruminococcaceae*, and *Faecalibacterium* were the obvious biomarkers; these representative microbiotas all contribute to the production of SCFAs [4,21]. LEfSe also highlighted the significant enrichment of *Desulfobacterota* and subordinate bacteria in NE-challenged laying hens with a diet of BEC. *Desulfobacterota* has been demonstrated to alleviate intestinal inflammation as a major source of propionic acid [51]. In the current study, a correlation analysis indicated that those various bacteria, including some previously discussed biomarkers, were significantly associated with SCFAs in cecal contents and intestinal redox balance mediators. *Fusobacterium*, *Phascolarctobacterium*, and *Rikenellaceae_RC9_gut_group* were positively correlated with acetic acid, butyric acid, and valeric acid, whereas Campylobacter was negatively associated with these SCFAs. Additionally, positive associations were found between *Shuttleworthia*, *Phascolarctobacterium*, and *Faecalibacterium* with jejunal Nrf2 and NQO1 expressions, while the association with jejunal MDA content was reversed. These intestinal microbiotas have been documented to have positive effects on intestinal redox homeostasis in previous research [54]. Conversely, *Escherichia-shigella* was positively correlated with intestinal oxidative stress, which is a common pathogen of intestinal diseases [55]. Therefore, the correlation analysis results further support the view that alterations in gut microbial communities motivated by dietary BEC supplementation may play a pivotal role in alleviating intestinal injuries caused by *C. perfringens* and coccidia. Taken together, our results estimate the impact of *C. perfringens* and coccidia and BEC on gut microbiota in laying hens, indicating that dietary BEC supplementation improves the composition and structure of the microbial community in laying hens challenged with *C. perfringens* and coccidia, thereby modulating the intestinal redox and microenvironmental homeostasis.

## 5. Conclusions

In conclusion, dietary supplementation with BEC effectively reduced the severity of intestinal and ovarian damage in laying hens challenged with *C. perfringens* and coccidia. The positive effects of BEC can be attributed to its ability to enhance histopathological changes and restore redox balance in the intestine and ovary by modulating the composition and structure of gut microbiota. These results indicate that BEC is a safe and effective feed additive, yielding beneficial effects on the intestinal and ovarian health of laying hens with NE. The underlying molecular mechanisms by which BEC modulates gut microbiota and impacts host immunity remain to be elucidated. In order to achieve optimum utilization of BEC supplementation in commercial poultry production, further investigation is required into its applicability across diverse breeds and in response to varying environmental conditions.

## Figures and Tables

**Table 1:** Effect of dietary BEC supplementation on jejunal and ovarian histopathology score of laying hens challenged with *C. perfringens* and coccidia.

Item ^1^	Jejunum	Ovary
CON	0.50^c^	0.25 ^a^
CC	4.50^a^	4.75 ^c^
BEC	0.38^c^	0.5 ^a^
BECC	3.25^b^	4.13 ^b^
SEM	0.23	0.19
*p*-value	<0.01	<0.01
Main effect *p*-value		
BEC	0.02	0.03
CC	<0.01	<0.01
CC*BEC	<0.01	0.33

^a, b, c^ Means with different superscripts within a column differ significantly (*p* < 0.05). ^1^ Each mean represents one layer/replicate, eight replicates/treatment. CON, control group; CC, *C. perfringens* and coccidia challenge; BEC, basal diet + 1000 mg/kg BEC; BECC, *C. perfringens* and coccidia challenge + 1000 mg/kg BEC.

**Table 2:** Effect of dietary BEC supplementation on the jejunal antioxidant capacity of laying hens challenged with *C. perfringens* and coccidia.

Item ^1,2^	SOD, U/mg	CAT, U/mg	GSH, U/mg	GSH-PX, U/mg	GSH-ST, U/mg	T-AOC, U/mg	MDA, nmol/mg
CON	104.35 ^b^	23.39 ^b^	37.31 ^b^	79.39	29.48 ^b^	1.21 ^ab^	1.22 ^b^
CC	432.57 ^a^	82.33 ^a^	88.46 ^a^	97.95	244.80 ^a^	2.31 ^a^	10.62 ^a^
BEC	89.94 ^b^	28.06 ^b^	19.65 ^b^	76.40	40.05 ^b^	0.95 ^b^	1.60 ^b^
BECC	276.45 ^ab^	43.68 ^ab^	45.56 ^b^	127.06	113.01 ^ab^	2.25 ^a^	5.31 ^b^
SEM	31.36	15.25	11.09	25.57	45.85	0.36	1.72
*p*-value	<0.01	0.05	<0.01	0.49	0.01	0.02	<0.01
Main effect *p*-value							
BEC	0.18	0.27	0.01	0.62	0.20	0.66	0.17
CC	<0.01	0.02	<0.01	0.19	<0.01	<0.01	<0.01
CC*BEC	0.26	0.17	0.27	0.54	0.14	0.79	0.11

^a, b^ Means with different superscripts within a column differ significantly (*p* < 0.05). ^1^ Each mean represents one layer/replicate, eight replicates/treatment. CON, control group; CC, *C. perfringens* and coccidia challenge; BEC, basal diet + 1000 mg/kg BEC; BECC, *C. perfringens* and coccidia challenge + 1000 mg/kg BEC. ^2^ SOD, superoxide dismutase; CAT, catalase; GSH, glutathione; GSH-PX, glutathione peroxidase; GSH-ST, glutathione S-transferase; T-AOC, total antioxidant capacity; MDA, malondialdehyde.

**Table 3:** Effect of dietary BEC supplementation on jejunal relative mRNA expression of laying hens challenged with *C. perfringens* and coccidia.

Item ^1,2^	Nrf2	Keap1	NQO1	HO-1	NF-κB	P53
CON	1.00 ^b^	1.00 ^a^	1.00 ^b^	1.00 ^ab^	1.00	1.00 ^bc^
CC	0.64 ^b^	0.39 ^b^	0.57 ^b^	0.63 ^b^	0.67	0.51 ^c^
BEC	2.10 ^a^	0.64 ^ab^	1.63 ^a^	1.27 ^a^	2.26	1.92 ^a^
BECC	0.69 ^b^	0.35 ^b^	0.68 ^b^	0.59 ^b^	0.69	1.36 ^ab^
SEM	0.22	0.18	0.15	0.14	0.55	0.27
*p*-value	<0.01	0.07	<0.01	<0.01	0.16	<0.01
Main effect						
*p*-value						
BEC	0.02	0.30	0.02	0.42	0.25	<0.01
CC	<0.01	0.02	<0.01	<0.01	0.09	0.06
CC*BEC	0.04	0.41	0.10	0.31	0.27	0.88

^a, b, c^ Means with different superscripts within a column differ significantly (*p* < 0.05). ^1^ Each mean represents one layer/replicate, eight replicates/treatment. CON, control group; CC, *C. perfringens* and coccidia challenge; BEC, basal diet + 1000 mg/kg BEC; BECC, *C. perfringens* and coccidia challenge + 1000 mg/kg BEC. ^2^ Nrf2, nuclear factor erythroid 2-related factor 2; Keap1, Kelch-like ECH-associated protein 1, NQO1, NAD(P)H: quinone oxidoreductase 1; HO-1, Heme oxygenase-1; NF-κB, nuclear factor-kappa-B.

**Table 4:** Effect of dietary BEC supplementation on ovarian antioxidant capacity of laying hens challenged with *C. perfringens* and coccidia.

Item ^1,2^	SOD, U/mg	CAT, U/mg	GSH, U/mg	GSH-PX, U/mg	GSH-ST, U/mg	T-AOC, U/mg	MDA, nmol/mg
CON	211.08	11.50	10.06	113.11	58.56	0.74	2.23 ^bc^
CC	217.69	7.26	15.29	98.09	54.48	2.21	3.86 ^ab^
BEC	219.49	9.27	9.41	106.68	50.82	0.96	1.63 ^c^
BECC	219.32	6.38	12.16	99.47	54.40	0.90	4.63 ^a^
SEM	13.42	1.45	2.47	8.14	6.56	0.53	0.71
*p*-value	0.97	0.09	0.35	0.54	0.87	0.21	0.02
Main effect *p*-value							
BEC	0.71	0.29	0.45	0.76	0.56	0.32	0.90
CC	0.81	0.02	0.12	0.19	0.97	0.20	<0.01
CC*BEC	0.80	0.64	0.62	0.64	0.56	0.16	0.35

^a, b, c^ Means with different superscripts within a column differ significantly (*p* < 0.05). ^1^ Each mean represents one layer/replicate, eight replicates/treatment. CON, control group; CC, *C. perfringens* and coccidia challenge; BEC, basal diet + 1000 mg/kg BEC; BECC, *C. perfringens* and coccidia challenge + 1000 mg/kg BEC. ^2^ SOD, superoxide dismutase; CAT, catalase; GSH, glutathione; GSH-PX, glutathione peroxidase; GSH-ST, glutathione S-transferase; T-AOC, total antioxidant capacity; MDA, malondialdehyde.

**Table 5:** Effect of dietary BEC supplementation on cecal alpha diversity of laying hens challenged with *C. perfringens* and coccidia.

Item ^1^	Observed Species	Shannon	Simpson	Chao1	ACE
CON	810.88 ^ab^	6.84 ^b^	0.98 ^a^	868.61	879.28
CC	747.50 ^b^	6.28 ^c^	0.95 ^b^	813.42	828.61
BEC	950.88 ^a^	7.24 ^a^	0.98 ^a^	1020.78	1034.1
BECC	840.75 ^ab^	6.59 ^bc^	0.97 ^a^	909.49	919.49
SEM	46.27	0.13	0.01	53.68	54.34
*p*-value	0.03	<0.01	<0.01	0.06	0.07
Main effect *p*-value					
BEC	0.02	0.01	0.03	0.02	0.03
CC	0.07	<0.01	<0.01	0.13	0.13
CC*BEC	0.62	0.73	0.45	0.60	0.56

^a, b, c^ Means with different superscripts within a column differ significantly (*p* < 0.05). ^1^ Each mean represents one layer/replicate, eight replicates/treatment. CON, control group; CC, C. perfringens and coccidia challenge; BEC, basal diet + 1000 mg/kg BEC; BECC, C. perfringens and coccidia challenge + 1000 mg/kg BEC.

## Data Availability

None of the data were deposited in an official repository. The dataset supporting those research conclusions are accessible on request.

## Supplementary Materials

The following supporting information can be downloaded at: https://www.mdpi.com/article/10.3390/ani15030299/s1, Table S1: Composition and nutrient level of basal diet (as-fed basis); Table S2: Sequences of real-time PCR primers.
