# Supplementary material for: Benzoic Acid, Enterococcus faecium, and Essential Oil Complexes Improve Ovarian and Intestinal Health via Modulating Gut Microbiota in Laying Hens Challenged with Clostridium perfringens and Coccidia
Source: Animals (Basel). 2025 Jan 21;15(3):299. doi: 10.3390/ani15030299 (PMC11816253; doi:10.3390/ani15030299)
Supplement: Supplementary file 1 [file animals-15-00299-s001.zip › animals-3409230-supplementary.pdf]

Table S1. Composition and nutrient level of basal diet (as-fed basis)

Item, %	Amount
Corn	55.15
Wheat bran	5.00
Soybean oil	3.00
Soybean meal（CP43%）	25.25
Stone powder (granular)	4.50
Stone powder (powder)	4.50
Calcium hydrogen phosphate (powder)	1.30
Sodium chloride	0.30
L-Lysine Sulfate (70%)	0.13
DL-methionine (99%)	0.20
L-threonine	0.09
Choline chloride, 60%	0.10
Vitamin premix[1]	0.03
Mineral premix[2]	0.45
Analyzed nutrient level	
Metabolizable energy[3], kcal/kg	2690
Crude protein, %	16.50
Crude fat, %	5.55
Crude fiber, %	2.71
Calcium, %	3.86
Total phosphorus, %	0.59
Lysine, %	0.85
Methionine, %	0.42

[1] Provided per kilogram of diet: vitamin A, 9300 IU; vitamin D_3, 3000 IU; vitamin E, 30 IU; vitamin K_3, 4.8 mg; vitamin B_1 (thiamine), 3 mg; vitamin B_2 (riboflavin), 9.6 mg; vitamin B_6, 6 mg; vitamin B_{12}, 0.3 mg; biotin, 1.67 mg; pantothenic acid, 18 mg; folic acid, 1.5 mg; niacin, 60 mg.

[2] Provided per kilogram of diet: copper ($CuSO_4 \cdot 5H_2O$), 8 mg; Iron ($FeSO_4 \cdot H_2O$), 60 mg; manganese ($MnSO_4 \cdot H_2O$), 60 mg; Zinc ($ZnSO_4 \cdot H_2O$), 80 mg; Iodine (KI), 0.35 mg; selenium (Na_2SeO_3), 0.3mg.

[3] Calculated according to NRC (1994).

Table S2. Sequences of real-time PCR primers.

Genes[1]	Orientation	Primer Sequences (5'-3')	Accession number
β-actin	Forward	ATCCGGACCCTCCATTGTC	NM_205518.1
	Reverse	AGCCATGCCAATCTCGTCTT	
Nrf2	Forward	TGTGTGTGATTCAACCCGACT	NM_205117.1
	Reverse	TTAATGGAAGCCGCACCACT	
Keap1	Forward	ACTTCGCTGAGGTCTCCAAG	XM_01527898.2
	Reverse	CAGTCGTACTGCACCCAGTT	
NQO1	Forward	GTTCAATGCCGTGCTCTCAC	NM_001277619.1
	Reverse	CCGCTTCAATCTTCTTCTGC	
OH-1	Forward	TTGCAAGAAGCATCCAGA	NM_2053344.1
	Reverse	TCCATCTCAAGGGCATTCA	
NFκB	Forward	GCTGCTTTGCACAGATGGA	NM_2205134.1
	Reverse	CCTTTGCAAAACTGGTTGGT	
P53	Forward	TACTCCCCGGTGCTGAATAA	NM_205264.1
	Reverse	GCCACGTGCTCTGATTTCTT	

[1] *Nrf2*: Nuclear factor-erythroid 2 related factor 2; *NQO1*: NAD(P)H: quinone oxidoreductase 1; *Keap1*: Kelch-like ECH-associated protein 1; *HO-1*: Heme oxygenase-1; *NF-κB*: nuclear factor-kappaB.
